# Cyanocobalamin improves memory impairment via inhibition of necrosis and apoptosis of hippocampal cell death after transient global ischemia/reperfusion

**DOI:** 10.22038/IJBMS.2020.48447.11126

**Published:** 2021-02

**Authors:** Hossein Khastar, Behzad Garmabi, Fatemeh Zare Mehrjerdi, Mohammad Taghi Rahimi, Nabi Shamsaei, Amir-Hossein Ali, Nilofar Khorsand, Mehdi Khaksari

**Affiliations:** 1 School of Medicine, Shahroud University of Medical Sciences, Shahroud, Iran; 2 Neurosciences Research Center, Shahroud University of Medical Sciences, Shahroud, Iran; 3 Neurobiomedical Research Center, Shahid Sadoughi University of Medical Sciences, Yazd, Iran; 4 Department of Sport Sciences, Ilam University, Ilam, Iran; 5 Student Research Committee, School of Medicine, Shahroud University of Medical Sciences, Shahroud, Iran

**Keywords:** Apoptosis, Brain ischemia, Cyanocobalamin, Hippocampus, Memory, Necrosis

## Abstract

**Objective(s):**

Brain ischemia/reperfusion (I/R) causes irreversible damage, particularly in the hippocampus. Cyanocobalamin (CNCbl) is known to be crucial for the proper operation of the nervous system. Vitamin B12 has been demonstrated to exert antioxidant effects via direct and indirect mechanisms. It can also protect cortical neurons against glutamate cytotoxicity. This research was conducted to examine CNCbl protection against neuronal cell death in the rat hippocampal region following transient cerebral ischemia.

**Materials and Methods:**

In this experiment, 48 male Wistar rats were selected, which were randomly divided into four groups (n=12 in each group): sham, ischemia/reperfusion, ischemia/reperfusion + CNCbl 200 and 400 (µg/kg). By occlusion of both common carotids, ischemia induction was performed within 20 min. CNCbl at the doses of 200 and 400 µg/kg was injected (IP) at the start of the reperfusion, 24 and 48 hr following reperfusion. The spatial memory was assessed 7 days following ischemia through the Morris water maze test. Antioxidant enzymes, apoptosis, and necrosis were measured after behavioral tests.

**Results:**

CNCbl significantly improved spatial memory impairments (*P*<0.05), also CNCbl therapy significantly increased both glutathione (*P*<0.01) and superoxide dismutase (*P*<0.05) and reduced malondialdehyde (*P*<0.01) and TNF-α (*P*<0.05) in comparison with the ischemia group. In addition, CNCbl significantly decreased both apoptosis and necrosis in the hippocampus CA1 (*P*<0.01).

**Conclusion:**

CNCbl improves memory impairment following ischemia injury by decreasing neuronal cell death via its antioxidant properties.

## Introduction

Brain ischemia has become one of the leading causes of mortality throughout the world (1). Intricate and dynamic alterations are caused by cerebral ischemia resulting in ischemic vascular damage and a degree of disruption in the blood-brain barrier (BBB). Research shows that sensitivity to cerebral ischemia is greater in some areas of the brain and some nerve cells such as hippocampal pyramidal neurons. (2, 3). Necrosis in the cells of the hippocampus can be observed following 6 min of ischemia (4). Extensive research has shown that the CA1 area of the hippocampus plays a key role in brain memory function, specifically in consolidation of the spatial memory information (5). 

Results from the recent studies investigating cerebral ischemia propose several new neuroprotective therapeutic approaches to decrease brain injury among animals (6). 

The neurons of the hippocampal CA1 area are selectively susceptible to transient global cerebral ischemia. Inflammation of the nervous tissue due to activation of the immune mediators after brain ischemia leads to neuronal cell death.

Cyanocobalamin (vitamin B12) has been shown to be crucial for normal performance of the neural system (7). methylcobalamin (MeCbl), Cyanocobalamin (CNCbl), adenosylcobalamin (AdoCbl), and hydroxycobalamin (OHCbl) are different analogs of vitamin B12 (8, 9). Vitamin B12 in the blood is transported by binding to transcobalamin, also it can enter the cell via its receptors (TC-R). Lysosomal membrane vitamin B12 transporter (LMBRD1) transfers vitamin B12 into the cytoplasm, in which it acts as a cofactor to Methylmalonyl-CoA mutase (MCM) as well as methionine synthase (MS). In addition, its deficiency results in methionine deficiency that is needed for phospholipids and myelin production (10, 11).

Older people are more likely to develop CNCbl deficiency (10 to 40%). CNCbl supplementation has been shown to be effective for treating inflammatory diseases including chronic fatigue syndrome, arthritis, sepsis, multiple sclerosis, and Alzheimer’s (12). Vitamin B12 also has anti-oxidant properties that exert their effects through both direct and indirect mechanisms. (11). It has been shown that in experimental diabetic neuropathy, continuous treatment of CH_3_-B12 in the peripheral nerve lesions is effective (13). Furthermore, vitamin B12 can possibly be used in several useful therapies to treat nervous diseases via beneficial systemic/local delivery of elevated concentration of MeCbl toward the considered areas (7).

CNCbl plays a protective role against the pathological effects of oxidative stress (14). This form of vitamin B12 can modulate the immune reactions and influence cytokine as well as growth factor synthesis. In this regard, the results obtained in one study showed that its deficiency leads to a reduction of the neurotrophic epidermal growth factor (EGF) production and elevation of neurotoxic cytokine tumor necrosis factor-α (TNF-α) synthesis (15, 16).

It has also been shown that NF-κB synthesis, through an unknown mechanism, can be suppressed by CNCbl (17). The protective effect of CNCbll against oxidative stress has been confirmed in cellular models. According to our literature review, no studies have yet elucidated the effects of CNCbl on reperfusion ischemia. Taken together, given the mechanisms of brain ischemia also protective impacts of CNCbl, the current study was designed to evaluate the impacts of CNCbl treatment on memory impairment and brain cell death following cerebral ischemia/reperfusion.

## Materials and Methods

Wistar rats weighing between 250 and 280 g were obtained from the Shahroud University of Medical Sciences, Iran. All rats were kept at 22 to 24 °C and 45 to 50% relative humidity under a 12 hr light/12 hr dark cycle (lights on = 08:00 am). Water and food were freely accessible. All experiments were carried out based on the Helsinki Declaration.

***Transient global cerebral ischemia model***

By using a procedure which was previously reported, transient global cerebral I/R injury model was induced in the following groups (n=12 per group): sham, I/R, I/R + cyanocobalamin 200/µg/kg and I/R + cyanocobalamin 400/µg/kg, cyanocobalamin intraperitoneally (IP) injection at first, 24, and 48 hr following reperfusion (18). Anesthesia was induced by ketamine/xylazine at 80 mg/kg by IP injection and then ischemia surgical procedure was carried out for animals. At the beginning of the study, bilateral carotid arteries were cautiously isolated from the vagus nerves and sheets, then arteries were occluded by using Yasargil aneurysm clips. After occlusion, the clips were separated from the arteries for restoring the blood flow and immediate reperfusion. The rectal temperature in animals was kept at 36.5±0.5 °C throughout the experiment for regulating the feedback of the heating system. Animals in the sham group were regarded as control subjects undergoing the same procedure, except for the carotid artery occultation. Following surgery, the rats were maintained separately for 7 days in home cages, where food and water were freely accessible (ethics code, IR.SHMU.REC.1396.124).

***Evaluation of the spatial memory***

The animals’ memory and spatial learning were measured 24 hr after the last injection by the Morris water maze (MWM) task. Four trials (20 min intervals) were considered to train the rats through four consecutive days. Animals had 60 sec for finding the hidden platform. We assessed the distance and time reaching the target quadrant that had a platform, using a camera followed by collecting the data by the software. After 24 hr of rest, their memory was assessed in the probe test. In brief, without the platform, the animals had 60 sec of swimming for finding the platform’s previous place in the target quadrant. The time elapsed in the target quadrant was measured (19).

***Biochemical assays***

Half of the rats (N=6) were anesthetized 40 days after birth by injection of xylazine/ketamine (10/80 mg/kg/IP) cocktail intraperitoneally (IP). First, immediately after anesthesia animal brains were gently harvested from the skull, then the hippocampal formation of each brain was isolated and homogenized in a lysis buffer (RIPA) with protease inhibitors. For the enzymatic assay, we used the supernatant after centrifugation at 3000 g (20 min, 4 °C).

***Evaluation of the concentration of malondialdehyde (MDA)***

In order to assess the degree of lipid peroxidation, an MDA kit was employed for measuring the concentration of MDA in the hippocampal samples. Using the TBARS assay method, MDA concentration was measured following tissue homogenization in 1.15% KCl (120 sec). The homogenized tissues were first mixed in thiobarbituric and trichloroacetic acid 10% (2 and 1 ml, respectively), and then heated for 60 min at one hundred degrees centigrade. Next, after cooling down and centrifugation of the solution, the precipitates were removed. Pink supernatant was then added to each well in the microplate. The reaction mixture absorbance was determined by employing a microplate reader set at 535 nm. Based on the standard curve, the level of MDA was measured (20).

***Catalase, superoxide dismutase (SOD), and glutathione (GSH) measurement***

Following the kit manufacturer’s instructions, the concentrations of GSH, catalase, and SOD were determined. By adding 100 mM PBS (pH 7.4), the samples were homogenized and centrifuged and then the supernatants were harvested. According to the previous methods, after the chromogenic reagent interaction, the total concentrations of GSH (420 nm), catalase (240 nm), and SOD (412 nm) were determined by using a BioTek microplate reader.

***Measurement of the TNF-α***

The level of TNF-α was determined based on the kit instructions (Diaclone). For coating the wells of microtiter strips, the specific monoclonal antibodies for TNF- α were applied. The samples (including unknown and known TNF-α level) were loaded into the wells and simultaneously incubated with biotinylated monoclonal antibody and TNF-α antigen. The streptavidin-peroxidase enzyme was added. To obtain a colored product, after incubation the unbound enzymes were discarded, and then a substrate solution was mixed in the bound enzyme. The product’s intensity was relative to the level of TNF-α. The results were expressed as pg/mg of protein (21).

***Tissue preparation***

Half of the rats in each group (n=6) were anesthetized by injection of chloral hydrate after behavioral test. By adding 4% paraformaldehyde and 0.9% saline to 0.1 M phosphate buffer, transcranial perfusion was performed. The animals’ brains after harvesting were embedded in the paraffin and postfixed for three days. According to the Paxinos atlas, the brain tissues were coronally cut using a microtome (19). 

***TUNEL staining***

According to the manufacturer’s protocol (In Situ Cell Death Detection Kit (Roche, Mannheim, Germany)), the TUNEL technique was used for determination of labeling DNA fragmentation (22). The tissue sections (3 sections per rat) were deparaffinized with xylol, rehydrated through descending alcohol series, and incubated at room temperature via proteinase K. 

In order to block endogenous peroxidase, the brain sections were formerly incubated with 3% hydrogen peroxide (H_2_O_2_) in a methanol solution. The TUNEL reaction mixture was then added in the wet atmosphere (37 °C). After performing a washing step, visualization was done using converter-POD for 30 min in the darkroom. The slides were then washed with DAB substrate (0.05% 3, 3-diaminobenzidine) and PBS for ten minutes. Afterward, hematoxylin was applied for counterstaining. Finally, counting the TUNEL positive cells was performed under a light microscope (LABOMED USA, magnification 400×) and during transect of the 400 µm length of the DG area of the right hippocampus. 

***Nissl staining***

Nissl staining was used to distinguish necrotic neurons from healthy ones in the brain (22). The brain sections (7 μm) were placed on the glass slides. The cresyl violet 1% (Sigma Aldrich) was used for staining, an investigator, who was blinded to the aim of the experiment counted three photomicrographs under a light microscope (400× magnification (Olympus AX-70)). The captured images were analyzed using the image tool 2 software. The number of cells was calculated along a transect (0.160 mm^2^,400 µm length) of the right hippocampal CA1 area (23).

***Data analysis***

The analysis of data was carried out using GraphPad Prism 6. One-way ANOVA analysis was employed for evaluation of the differences between the animal groups, followed by Bonferroni and Tukey’s *post-hoc* tests and two-way repeated-measures ANOVA. The results were presented as mean±SEM, and *P*≤0.05 were considered statistically significant.

## Results

***CNCbl effect on spatial learning and memory impairment following brain ischemia***

**Figure 1:**
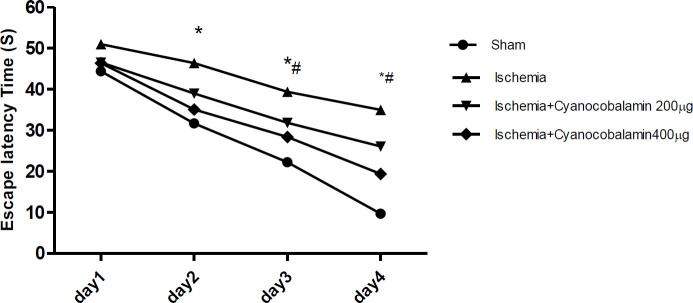
Escape latency time of rats searching for the hidden platform over 4 consecutive training days in the MWM task for all groups

**Figure 2:**
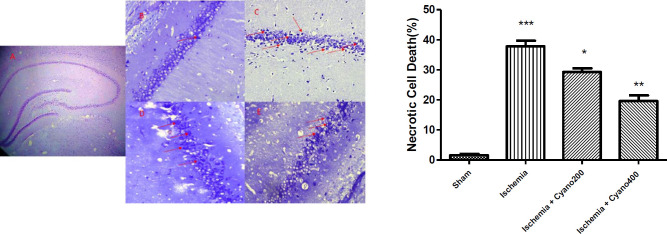
Spending time in the target zone (right) and velocity in probe day (left)

**Figure 3:**
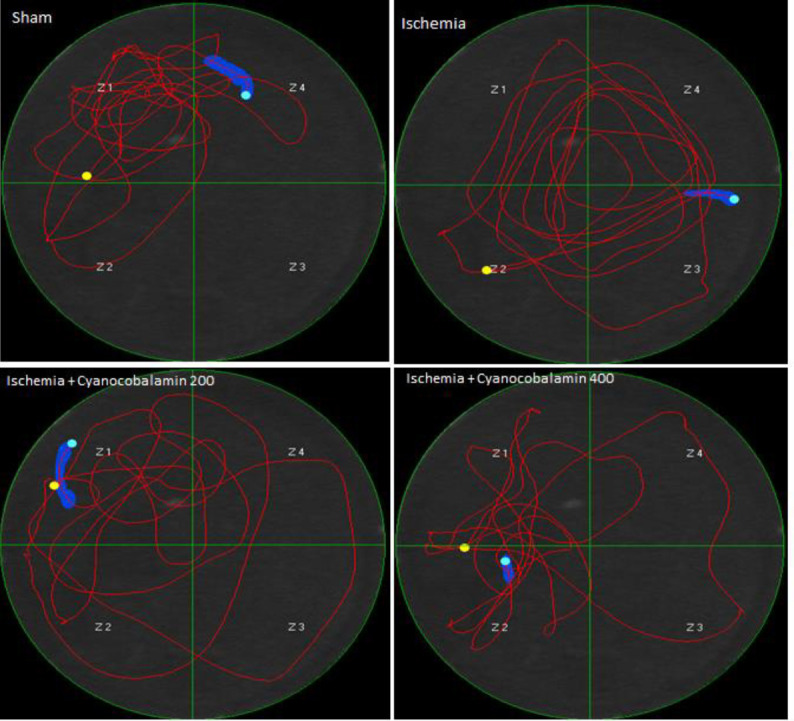
Swimming paths in the MWM task on the fifth day of the experiment (probe trial day) for different groups

Examination of the escape latency to find the platform in the trial days showed that there is a significant difference between the groups. The data indicated that the ischemic group spent more time to find the hidden platform on experimental days, in comparison with the sham group (*P*<0. 001, Figure 1). The distance and time for reaching the hidden platform on all test days significantly decreased in ischemia rats treated with CNCbl, compared with the ischemia group (*P*<0.01). Probe data showed that the sham group spent more time in the target quadrant than the ischemic group (*P*<0.001, Figures 2 and 3). Spending time in the target quadrant was significantly increased in the ischemic CNCb1 treated group (*P*<0.05). The swimming velocity of animals was tested on probe and training days to rule out the possibility that the above consequences are due to impaired motor functioning. The results demonstrated that there was no significant difference between groups, which means that the animal motor functions were not affected by ischemia (Figure 2).

***CNCbl increased SOD and GSH levels after ischemia***

**Figure 4:**
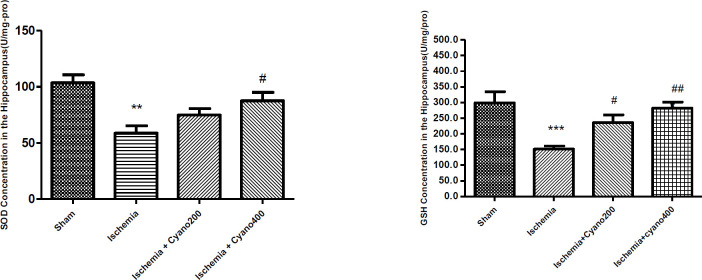
Effects of cyanocobalamin (200, 400 µg) on the levels of GSH and SOD in the hippocampus following I/R, (N=6 per group)

In comparison with the sham group, the concentration of SOD was significantly reduced in the ischemia group (*P*<0.01). In ischemic rats on CNCbl treatment, SOD levels increased compared with the ischemic rats (*P*<0.05). GSH levels in the ischemia group were decreased compared with sham (*P*<0.001). In ischemic rats on CNCbl treatment, the concentration of GSH significantly increased compared with the ischemic rats (*P*<0.01, Figure 4). 

***CNCbl decreased the concentration of MDA and TNF-α after ischemia***

**Figure 5:**
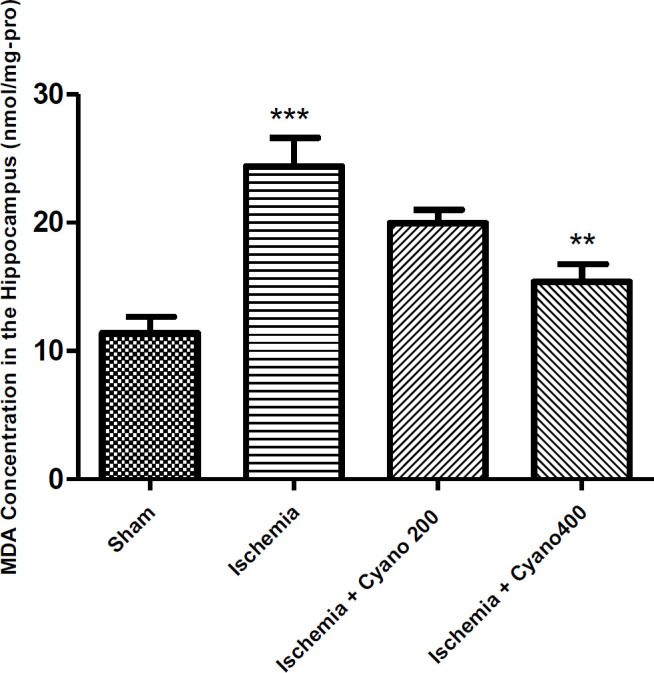
Effects of cyanocobalamin (200, 400 µg) on MDA concentration in the hippocampus following I/R, (N=6 per group)

**Figure 6:**
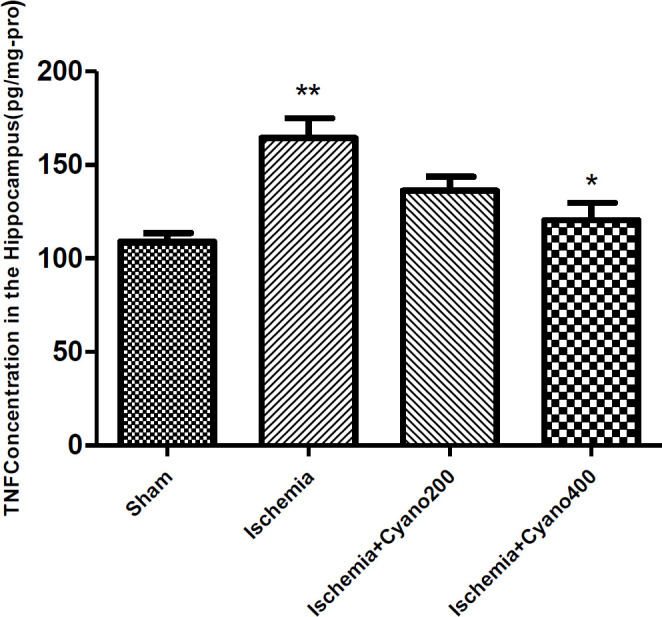
Effects of cyanocobalamin (200, 400 µg) on the level of TNF-α in the hippocampus following I/R, (N=6 per group)

Biochemical assessment of MDA levels in the hippocampus showed that the level of this organic compound significantly increased in the ischemic group, compared with the sham group (*P*<0.001). In comparison with the ischemic group, the level of MDA was significantly decreased following treatment with CNCbl (*P*<0.01, Figure 5). The concentration of TNF-α in the hippocampus of ischemic animals was higher than in the sham animals (*P*<0.01). CNCbl treatment reduced the level of TNF-α versus the ischemic group (*P*<0.05, Figure 6).

***CNCbl prevents ischemia-induced apoptosis cell death***

**Figure 7:**
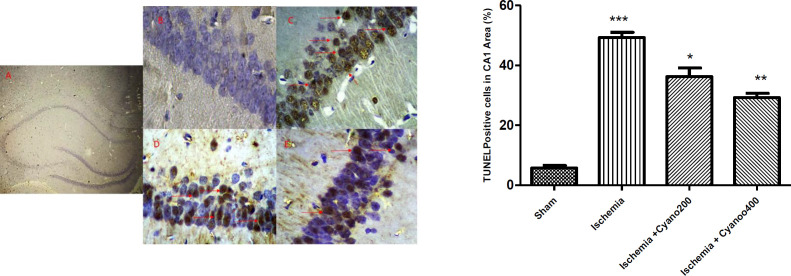
Effects of cyanocobalamin on the percentage of TUNEL positive cells. Photomicrographs of TUNEL staining in the hippocampal CA1 region after I/R. A: Hippocampus section B: Sham, C: Ischemic group, D: Ischemic group+cyanocobalamin 200 µg, E: Ischemic group+cyanocobalamin 400 µg, (magnifications ×400). Red arrows show apoptotic cell death

In the right hippocampal CA1 region, TUNEL-positive cell number was higher in the ischemic group than in the sham one (*P*<0.001). Nevertheless, the percentage of TUNEL-positive cells in the ischemic/CNCb1 group was significantly lower than in the ischemic group (*P*<0.01, Figure 7). 

***CNCbl weakened ischemia-induced necrosis cell death***

**Figure 8:**
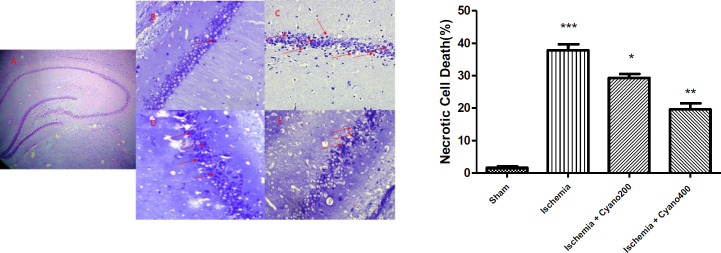
Effects of cyanocobalamin on necrotic cell death following I/R in the right hippocampal CA1 region. Cyanocobalamin treatment significantly decreased I/R-induced necrotic cell death

Observation of the Nissl-stained photomicrographs showed that the number of necrotic cells in the right hippocampal CA1 region was significantly increased in the ischemic group, compared with the sham group (*P*<0.001). In ischemic rats that were treated with CNCbl, necrotic cells were significantly (*P*<0. 01, Figure 8) decreased versus ischemic animals.

## Discussion

For the first time, the current study indicated that the CNCbl is able to significantly improve memory impairment, also reduce apoptotic and necrotic cell death following brain ischemia/reperfusion in the CA1 region of rats’ hippocampus. In addition, based on the findings, CNCbl is beneficial to reduce TNF-α production and inhibit lipid peroxidation, also significantly increase anti-oxidant enzyme (GSH and SOD) levels after brain ischemia. 

The underlying pathophysiology mechanisms of cerebral ischemia/reperfusion are very complicated. Reperfusion can affect brain damage and cause some processes after blood flow restoration, including oxidative stress, lack of energy, inflammation, excitotoxicity, impairment in regulation of calcium, and activation of cell signaling pathways such as apoptosis (24, 25). Any factor inhibiting such processes can be useful to treat brain ischemia.

The role of reactive oxygen radicals (ROS) in brain damages, such as cerebral I/R, has been shown in many studies. Cerebral blood flow (CBF) decreased after focal or global cerebral ischemia, whereas the occluded vessels are needed to provide blood in such areas. Oxygen, as a very important substrate in several enzymatic responses, can be provided via reoxygenation/ thrombolytic reperfusion in the cytosolic or subcellular organelles as well as mitochondria. It has been shown that SOD, glutathione peroxidase (GSH-Px), and catalase are able to scavenge ROS.

Neuronal cell death is affected by elevated reactive nitrogen species and ROS due to protein oxidation, DNA damage, and enhanced lipid peroxidation through cell membrane (26, 27). SOD and GSH-Px activities can be increased by CNCbl. So, the level of DNA damage is also reduced. MDA as a compound with cytotoxic activity is generated via lipid peroxidation and is also the biomarker for oxidative stress. It is indicative of free radical synthesis and damage in subsequent tissue (28). SOD and GSH as anti-oxidant enzymes are protective against oxidative insult. They are needed for defense strategies and thus the survival of aerobic organisms. The current study results showed that the levels of GSH and SOD significantly decreased the plasma anti-oxidant effect after I/R, however, an increase in the level of MDA was consistent with the results of other studies (29). One of the possible neuroprotective mechanisms of CNCbl can be result of its neurotropic action in the CNS which is caused by stimulating the synthesis of EGF.

Research shows that EGF may be produced by neural cells, such as hippocampal neurons (30) and the same is true for EGF receptors. Although both the EGF and its receptors are produced by hippocampal cells, there is no evidence to show that EGF can act on neurons in an autocrine manner. Experimental research shows that EGF synthesis is low through the hippocampal formation, while the expression of its receptors is high in this area of the brain (31, 32). 

*In vitro* studies have shown that EGF is a neuroprotective compound. It is also reported that EGF protects neurons in the hippocampus against ischemia-related damage *in vivo*. (33). Previous studies demonstrated that CNCbl caused an increase in EGF synthesis (34, 35).

Proinflammatory pathways that are activated due to brain ischemia-reperfusion are characterized by TNF-α and interleukin-1β (IL-1β) synthesis (36). Therefore, inhibition of these inflammatory mediators is a good candidate to alleviate the brain injury induced by I/R. Previous studies showed that TNF-α induces direct neuronal toxicity via ROS production by the secondary messenger ceramide for causing neuronal apoptosis (37). Previous study illustrated that CNCbl deficiency is associated with elevated levels of TNF-α, so treatment with CNCbl can have anti-inflammatory effects by lowering TNF-α levels (16).

Expression of NF-κB has been shown in several types of neuronal cells, and it is generally active at relatively low concentrations in the cortex and hippocampal rodents’ brain cells (38). Although experimental studies have shown inducible NF-κB-activity in neurons, like glutamate stimulation or seizure activity, its functional importance has not yet been identified (39). Several stimuli including glutamate, IL-1α, hypoxia, TNFα, and reactive oxygen species seen in ischemia can activate NF-κB (40, 41). In cerebral ischemia, NF-κB secretion is associated with neuronal cell death. Previous studies demonstrated that CNCbl reduces the level of NF-κB (17, 42), so one of the mechanisms of CNCbl neuroprotection is probably modulation of NF-κB expression.

* vs sham group (*P*<0.001); # vs ischemic group (*P*<0.01)

*** vs sham group (*P*<0.001); *vs ischemic group (*P*<0.05)

*** vs sham group (*P*<0.01); ** vs sham group (*P*<0.01); * vs ischemic group (*P*<0.05); ## vs ischemic group (*P*<0.01); # vs ischemic group (*P*<0.05)

*** vs sham group (*P*<0.001); ** vs ischemic group (*P*<0.01)

** vs sham group (*P*<0.01); * vs ischemic group (*P*<0.05)

*** vs sham group (*P*<0.001); ** vs ischemic group (*P*<0.01); * vs ischemic group (*P*<0.05)

Photomicrographs of Nissl staining in the CA1 area of the hippocampus after I/R. A: Hippocampus section B: Sham, C: Ischemic group, D: Ischemic group+cyanocobalamin 200 µg, E: Ischemic group+cyanocobalamin 400 µg, (magnifications ×400). Red arrows show necrotic cell death

*** vs sham group (*P*<0.001); ** vs ischemic group (*P*<0.01); * vs ischemic group (*P*<0.05)

## Conclusion

The current study showed that CNCbl can improve memory dysfunction and reduce neuronal injury in rat’s hippocampal CA1 region due to cerebral ischemia. CNCbl neuroprotective impact can be due to several mechanisms, including apoptosis and necrosis inhibition, as well as improved anti-oxidant system. Considering CNCbl protection against pathological conditions, this medication can be suggested as an appropriate therapeutic candidate for several disorders like cerebral I/R.
